# Measuring character strengths as possible protective factors against suicidal ideation in older Chinese adults: a cross-sectional study

**DOI:** 10.1186/s12889-020-8457-7

**Published:** 2020-04-03

**Authors:** Xinfeng Cheng, He Bu, Wenjie Duan, Along He, Yaping Zhang

**Affiliations:** 1 Economics and Management Department, Xi’an Technological University, Xi’an, China; 2 School of Public Policy and Administration, Xi’an Jiaotong University, Xi’an, China; 3 Department of Social and Behavioural Sciences, City University of Hong Kong, Hong Kong, China; 4 Social and Public Administration School, East China University of Science and Technology, Shanghai, China; 5 Department of Sociology, Wuhan University, Wuhan, China; 6 Goizueta Business School, Emory University, Atlanta, Georgia

**Keywords:** Character strengths, Life satisfaction, Psychometric, Suicidal ideation, China

## Abstract

**Background:**

Suicide is a global issue among the elderly. The number of older people committing suicide is proliferating, and the elderly suicide rate is the highest among all age groups in China. A better understanding of the possible protective factors against suicidal ideation is necessary to facilitate prevention and intervention efforts. The objectives of the present study are threefold. First, this study aims to examine the psychometric properties of the three-dimensional inventory of character strengths (TICS) with a sample of older adults. Second, this study intends to investigate correlations among suicide ideation, wellbeing, and character strengths. Third, the study seeks to explore the possible protective roles of the three character strengths and wellbeing in explaining suicidal ideation among older adults.

**Methods:**

A cross-sectional study comprising 308 older adults aged at least 50 years old from nursing homes was conducted. Four questionnaires, namely, the TICS, the Geriatric Suicide Ideation Scale—10 items, the Brief Inventory of Thriving, and the Satisfaction with Life Scale, were used. Exploratory structural equation modeling, intraclass correlation coefficients, partial correlations, and sets of hierarchical regressions were adopted to estimate and report the results.

**Results:**

TICS could be used to assess the character strengths (i.e., caring, inquisitiveness, and self-control) among older adults with an acceptable goodness-of-fit (chi square = 157.30, *df* = 63, *p* < 0.001, CFI = 0.94, TLI = 0.90, RMSEA = 0.07, 90% CI = [0.06, 0.08]). Wellbeing and character strengths exhibited a negative association with suicidal ideation among older adults. Moreover, character strengths showed an independently cross-sectional relationship with suicidal ideation, explaining 65.1% of the variance of suicidal ideation after controlling for the wellbeing and demographics.

**Conclusion:**

This study indicated that character strengths were associated with low levels of suicidal ideation. Therefore, the protective factors against suicidal ideation among older adults should be given additional attention.

## Background

The cross-national patterns of suicide rates, which can be influenced by gender and other factors, differ. Nevertheless, suicide rates generally increase with age. Since 1987, when China’s suicide mortality statistics initially became available from the Ministry of Health, suicide rates in older adults have always been higher than those in young adults and in other countries [1]. As the population in China ages, the number of older people also increases; hence, suicide rates can worsen. Therefore, an improved understanding of this phenomenon is necessary to facilitate prevention and intervention efforts.

Previous studies have focused on the risk factors of suicidal ideation among older adults. From the interpersonal-psychological theory of suicide [2, 3], two active psychological constructions, that is, thwarted belongingness and perceived burdensomeness, predict suicidal ideation. Thwarted belongingness refers to social alienation, whereas perceived burden occurs when older adults regard themselves as a burden to others, thus potentially generating self-hatred [4]. The interpersonal-psychological theory explains a series of risk factors for suicidal ideation [5]. Many other previous studies in this area have demonstrated that these risk factors, such as the older adults’ age, financial strain, poor health, depression, filial piety of children, social network, low perceived control, and loneliness, are correlated with suicidal ideation [1]. For instance, negative emotions and the lack of positive ones are highly correlated with suicidal ideation and can reduce the hope of older adults to lead a productive life [6]. A sense of loneliness may stimulate a high degree of threat surveillance, ultimately reducing executive functioning and weakening an individual’s ability to participate in healthy behaviors required for self-control, such as regular exercise [7]. Depression and hopelessness are proven markers of increased suicidal ideation in older adults [8]. Negative events in the family, such as quarrels and illness and death of family members and friends, are also closely relevant to suicidal ideation [9]. Older adults, who isolate themselves from society, feel lonely, and worry about their future, may come up with the idea of ending their life to stop their suffering. These studies have indicated that negative individual (e.g., hopelessness) and social (e.g., isolation) factors can contribute to the risk of suicidal ideation of older adults. Recently, with the emergence of positive psychology, which has been defined as the scientific study of optimal human functioning [10], an increasing number of researchers has shifted their focus from passive influential factors to the effect of positive factors [11].

Protective factors are conditions or attributes (e.g., living environment, living conditions, skills, strengths, resources, personal character, supports, or coping strategies) that help in the prevention or reduction of suicide ideations and suicidal behaviors. An improved understanding of these factors can help health care providers and mental health professionals to prevent suicidal ideation and enhance protective factors via interventions. Several protective factors, such as a sense of belongingness [12], reasons for living, and perceived social support [9], may lessen suicidal ideation among older adults. For instance, hope is a robust protective factor against suicidal ideation [13]. Researchers observed that hope is a sign of decreased perceived burdensomeness and thwarted belongingness and can play a role against hopelessness when dealing with suicidal ideation [14]. Moreover, after suffering from bereavement, which reduces the wellbeing of older adults, a heightened social trust can reduce the difficulty of the situation for older adults and enable them to find social companionship aside from their family [15]. Therefore, trusting social interaction can have a significant influence on preventing older adults from withdrawing into themselves day by day and reducing loneliness and suicidal ideation [16].

In positive psychology, two major dimensions contribute to mental health, that is, wellbeing and character strength. Wellbeing is a multidimensional construct [17] that includes subjective wellbeing (e.g., satisfaction with life) and psychological wellbeing (i.e., thriving). Previous studies have identified that decreased wellbeing is linked to high suicidal ideation [2, 5]. Cross-sectional, longitudinal, and experimental data have also indicated that wellbeing can lead to diverse positive outcomes on mental health [18].

In addition, character strengths are positive patterns embodied in emotions, thoughts, and behaviors that facilitate personal development and are universally valued [19]. Peterson and Seligman proposed the Values in Action Inventory of Strengths (VIA-IS) to assess 24 character strengths categorized under six virtues [19]. However, studies indicated that this English questionnaire had difficulty in converting factor structures among various cultures and countries. Thus, the model relating six higher-order virtues of 24 character strengths may be inconsistent across cultures [20–22]. Later, studies found the three-dimensional model of character strengths as a reliable solution to character strength structure [23, 24]. The three character strengths are caring (i.e., show of love, care, and gratitude for others), inquisitiveness (i.e., curiosity and passion for creativity), and self-control (i.e., quality of persistence and self-control). The three-dimensional inventory of character strengths (TICS) was developed and validated [25, 26] and was successfully applied in various populations, such as undergraduates, community participants, and inpatients [24, 27]. This model shows consistency with Asian and Western populations and among people of different gender, age, educational attainment, and marital status categories [27]. However, the new instrument, TICS, has not been applied in our target population, the elder population. Hence, this study aimed to determine whether TICS is applicable to older people.

Cross-sectional studies have indicated that character strengths are associated with positive mental health outcomes, such as life satisfaction and happiness, wellbeing, positive emotions, quality of life [28, 29], low levels of perceived life stress, and a meaningful life [30, 31]. One study determined that character strengths were associated with reduced suicidal ideation among students [32]. Therefore, the present study aims to apply TICS to older adults and further examine the protective role of character strengths against suicidal ideation. The current research is based on and ensures the continuity of the previous research. In addition, this study further examines if character strengths exhibit an interdependent association with suicidal ideation beyond the influence of wellbeing.

In summary, this study attempts to explore the relationships between wellbeing and character strengths on suicidal ideation among Chinese older adults from the perspective of positive psychology. The aims are twofold. First, this work examines the psychometric properties of TICS with a sample of older adults to expand the applicability of this newly developed instrument. The valid instrument for the elderly can be helpful in future character strength-based interventions. For example, practitioners can use the TICS to help participants identify their signature character strengths, which is one of the commonly used strategies in character strength-based interventions [33]. Second, relationships between suicidal ideation and character strengths, in conjunction with two dimensions of wellbeing (i.e., life satisfaction and thriving), are explored. This study further examines whether character strengths could exhibit an independent association with suicidal ideation beyond the influence of wellbeing and demographics. We hypothesized that after adjusting for demographic variables, wellbeing and character strengths show a significantly negative association with suicide ideation among Chinese older adults. Furthermore, character strengths exhibit a significant additional variance in suicide ideation scores after accounting for the wellbeing.

## Methods

### Participants

A total of 308 participants (female: *N* = 151, male: *N* = 157; mean age = 73.06 years, standard deviation [SD] = 8.75 years, range = 53–91 years) were recruited from nine public nursing homes from nine districts in the city of Chongqing, China.

### Sampling and recruitment

Nursing homes have become an alternative residence not only for frail older people but also for the broader elderly population to spend their old age [34]. Therefore, we left the recruitment information in nursing homes to reach our target population. An opportunity sampling method was used to recruit participants in this study. The staff helped in asking older adults if they were interested in participating in the research. Data were collected from March 2017 to May 2018. The eligibility criteria for participants included the following: (a) at least 50 years old; (b) volunteers to complete the questionnaires; (c) in good physical and mental health; (d) cognitive fitness and sufficient education to understand the questionnaire clearly. Older adults were ineligible if they have a cognitive impairment and a clinical diagnosis of mental illness. The exclusion criteria were assessed through prescreening conducted by well-trained and seasoned staff working in the nursing home. The older adults, who presented circumstances (e.g., dementia, stroke, and cerebral palsy) in which they could not complete the survey, would be referred to special care and were excluded from our study. Whether the subjects had suicidal ideation was not included in the recruitment criteria. Eligible participants were referred to our research team to complete the questionnaires.

### Study procedure

Participants were invited to attend a 30-min paper-and-pencil test comprising four questionnaires, which is presented in three blocks (one questionnaire in each block, except for the wellbeing questionnaires that were presented together in one block). The first, second, and third blocks contained 15 items that assess character strengths, 10 items that measure suicidal ideation, and 15 items that evaluate wellbeing, respectively (see the “Measures” section for details on each questionnaire). Participants were given a 3–5-min rest after completing each block to reduce fatigue and cognitive load. Demographic information (e.g., gender and age) was collected after they completed the questionnaire. All questionnaires, including the demographic information, were self-reported. Two social workers and a Master of Social Work student assisted the older people with poor eyesight and those that may have misunderstood the questionnaires in answering. They supervised the tests and provided prompt instructions to participants during the tests. No inducement was involved in the process. We did not provide the participants with any compensation or incentive. This study was conducted in Chinese, and all the materials used in the study (e.g., the recruitment information and questionnaires) were presented in Chinese.

### Measures

#### TICS

TICS is a brief, newly developed scale for measuring character strength, that is, caring (e.g., “Respect for the group’s decision is important to me.”), inquisitiveness (e.g., “I can always come up with new ways to do things.”), and self-control (e.g., “I am a highly self-disciplined person.”), in diverse populations [24]. The three factors of character strength have been verified in previous studies [21, 23, 27]. The current 15-item TICS showed sound psychometric characteristics among westerners, easterners, community population, and medical samples. Participants were required to rate each item from 1 (“very much unlike me”) to 5 (“very much like me”). Subscale scores were obtained by averaging across items in the scale.

#### Geriatric suicide ideation scale—10 items (GSIS–10)

The GSIS–10 is a subscale of the Geriatric Suicide Ideation Scale (GSIS), which is commonly used to measure suicidal ideation among old adults (e.g., “Hope my life will end soon”). GSIS is a 31-item scale used to assess in four dimensions (i.e., suicide ideation, death ideation, loss of personal and social worth, and perceived meaning in life) [35]. The 10-item suicide ideation subscale measured in one dimension, that is, suicidal ideation and differentiated psychiatric patients from non-patients. Participants were asked to rate the extent to which they agreed with each item using a five-point Likert scale (1 = “strongly disagree” to 5 = “strongly agree”). High mean scores indicated high suicidal ideation. The reliability and validity of the Chinese version of GSIS were confirmed [36].

#### Brief inventory of thriving (BIT)

The BIT comprises 10 important personal life facets (10 items) that determine the level of thriving and is a comprehensive measure of wellbeing (e.g., “I am optimistic about my future.”) [37]. A previous study evaluated the psychometric properties of BIT in China [38]. A total of 705 community participants, including older adults, completed the questionnaire and presented evidence of the Chinese version of BIT: a good internal consistency (Cronbach’s alpha > 0.85), a solid factor structure (*χ*^2^ = 60.35, *df *= 35, CFI = 0.96, TLI = 0.95, RMSEA = 0.05, 90% CI = [0.03, 0.06]), and good convergent (i.e., positively related to life meaning and positive affect, *r* = 0.34–0.65, *p* < 0.01), discriminant (i.e., negatively related to psychopathology symptoms, *r* = − 0.23 to − 0.48, *p* < 0.01), and incremental validities (i.e., additionally contributed 7.00% variance to psychopathology symptoms after controlling for wellbeing). Accordingly, all of these facets of BIT are pertinent to older adults in China, including those who live in nursing homes. In this study, the participants were asked to respond to each item using a five-point Likert scale (1 = “strongly disagree” to 5 = “strongly agree”).

#### Satisfaction with life scale (SWLS)

Diener and Emmons constructed this widely used five-item self-reported scale that measures life satisfaction (e.g., “In most ways, my life is close to my ideal.”) [39]. SWLS measures one dimension of wellbeing and is a cognitive evaluation or judgment of one’s life. The participants were asked to answer each item using a seven-point Likert scale (1 = “strongly disagree” to 7 = “strongly agree”). Previous studies showed that the coefficient alpha (0.87) and test–retest coefficient (0.82) were relatively high in various populations [40].

### Data analysis plan

All pieces of questionnaires have been double-checked with the help of the social workers and staff of the nursing homes to guarantee the quality of data input. The dataset of the current study has no missing item and data.

The data analysis plan had three steps. First, an exploratory structural equation modeling (ESEM) was conducted to examine the three-factor structure of TICS among older adults. In this study, the comparative fit index (CFI), the Tucker–Lewis Index (TLI), and the root mean square error of approximation (RMSEA) with its 90% confidence interval were examined. Brown noted that CFI > 0.95, TLI > 0.95, and RMSEA < 0.05 or 0.08 suggest an adequate and excellent fit to the data [41].

Second, the intraclass correlation coefficients (ICC) were used to indicate the reliability of all measures in the current sample, which could evaluate the 95% confidence interval where the true ICC value lands on [42]. Cronbach’s alpha is a special application of ICC [42, 43], but it may provide a biased impression of the poor when the number of the items decreased [44]. Therefore, we used ICC parameters with the 95% confidence interval in the current study, which has been regarded as a desirable index of the level of reliability [42]. In the current study, the “two-way random-effects model” and “consistency” type of ICC form were adopted to calculate the ICC parameters [42]. Koo and Li [42] noted that the ICC values less than 0.50, between 0.50 and 0.75, and 0.75 and 0.90, and greater than 0.90 are indicative of poor, moderate, good, and excellent reliability, respectively.

Third, the descriptive statistics and partial correlations among character strengths, suicidal ideation, and wellbeing were calculated with gender and age as the control variables. The correlations between character strengths and wellbeing were expected to be positive, whereas those between character strengths and suicidal ideation were expected to be negative. Fourth, sets of hierarchical regressions using the entry method were performed to explore the possible protective roles of the three character strengths and wellbeing in explaining suicidal ideation among older adults, with the adjustment of two demographic variables (i.e., gender and age). Suicidal ideation was set as the dependent variable in the hierarchical regression. The two demographic variables (i.e., gender and age) were used in Step 1, followed by two wellbeing indicators in Step 2. Then, the three character strengths were included in Step 3. This exploratory investigation would show the relative roles of character strengths and wellbeing in explaining suicidal ideation among older adults. SPSS 24.0 and Mplus 7.4 were used to conduct the aforementioned analyses.

## Results

### ESEM analysis

The three-factor correlated model TICS exhibited an acceptable goodness-of-fit (chi square = 157.30, *df* = 63, *p <* 0.001, CFI = 0.94, TLI = 0.90, RMSEA = 0.07, 90% CI = [0.06, 0.08]). Furthermore, the standardized parameter estimates from this ESEM model, reported in Table 1, showed that the TICS model held a clear factor structure. These estimates showed that items 1, 2, 4, 6, and 10 loaded prominently on the self-control factor, items 3, 5, 7, 8, and 9 on inquisitiveness factor, and items 11, 12, 13, 14, and 15 on caring factor. All were consistent with its intended factor loadings.

**Table 1:** Standardized parameter estimates from the ESEM of TICS

Item	F1 (λ)	F2 (λ)	F3 (λ)	δ
1. I am a highly disciplined man.	−0.131	0.010	**0.717**	0.540
2. I always think before I talk.	0.019	−0.218	**0.711**	0.531
3. I have the ability to make other people interested in something.	0.009	**0.485**	0.096	0.725
4. I’m a real lifelong learner.	−0.060	0.177	**0.598**	0.577
5. I can always think of new ways to do things.	−0.088	**0.458**	0.365	0.592
6. I don’t give up.	0.039	0.339	**0.445**	0.576
7. I never let the depressed situation take my sense of humor away.	0.135	**0.662**	−0.031	0.519
8. I’m full of energy.	0.005	**0.634**	0.101	0.548
9. In any case, I can always find fun.	0.100	**0.698**	−0.003	0.473
10. Deliberation is one of my characteristics.	0.082	−0.050	**0.646**	0.552
11. I enjoy the feeling of being good to others.	**0.568**	0.179	0.013	0.593
12. It is important for me to respect the decision of the group.	**0.606**	0.103	0.005	0.592
13. I think everyone should have the right to speak.	**0.877**	−0.028	−0.018	0.253
14. As a group leader, I think each member has the right to comment on what the group does.	**0.857**	0.004	−0.009	0.271
15. People believe I can keep their secret.	**0.499**	0.182	0.091	0.624

Note. λ standardized factor loading, δ standardized item uniqueness, *FI* Caring, *F2* Inquisitiveness, *F3* Self-control

### Reliability

The reliability of TICS in the current sample was acceptable, with ICC = 0.69 [95% CI = 0.63–0.74] for self-control, ICC = 0.62 [95% CI = 0.55–0.68] for inquisitiveness, and ICC = 0.77 [95% CI = 0.73–0.81] for caring. The GSIS in the older adults showed a moderate to good reliability with ICC = 0.75 [95% CI = 0.71–0.79]. The reliability of BIT was moderate (ICC = 0.69, 95% CI = 0.63–0.74]), and the reliability of SWLS was moderate to good (ICC = 0.76, 95% CI = 0.72–0.80).

### Descriptive and correlation analysis

Table 2 shows the means and SDs of the three character strengths, suicidal ideation, and wellbeing. The three strengths of character, caring, self-control and inquisitiveness, showed negative correlations (*r* = − 0.54, − 0.56, and − 0.44, respectively, *p* < 0.001) with suicidal ideation. Moreover, suicidal ideation was negatively related to thriving (*r* = − 0.59, *p* < 0.001) and wellbeing (*r* = − 0.41, *p* < 0.001). However, age and gender exhibited an insignificant correlation (*r* = −0.05 and − 0.01, *p* = 0.37 and 0.83, respectively) with suicidal ideation. Furthermore, the three character strengths were positively related to the wellbeing measures (*r* = 0.28 to 0.53, *p* < 0.001).

**Table 2:** Descriptive and correlations (*N* = 308)

		1	2	3	4	5	6	7	8
1 Age	Pearson’s r	–	0.07	0.06	−0.03	0.00	0.03	0.01	−0.05
2 Gender	Pearson’s r		–	0.00	0.06	−0.05	−0.02	− 0.01	−0.01
3 Self-control	Pearson’s r			**0.69 [0.63–0.74]**	0.14*	0.12*	0.35***	0.53***	−0.56***
	Partial r			–	0.15*	0.12*	0.35***	0.53***	−0.56***
4 Inquisitiveness	Pearson’s r				**0.62 [0.55–0.68]**	0.10	0.45***	0.48***	−0.44***
	Partial r				–	0.10	0.45***	0.49***	−0.44***
5 Caring	Pearson’s r					**0.77 [0.73–0.81]**	0.28***	0.38***	−0.54***
	Partial r					–	0.28***	0.38***	−0.54***
6 Life Satisfaction	Pearson’s r						**0.76 [0.72–0.80]**	0.53***	−0.41***
	Partial r						–	0.53***	−0.41***
7 Thriving	Pearson’s r							**0.69 [0.63–0.74]**	−0.59***
	Partial r							–	−0.59***
8 Suicidal ideation	Pearson’s r								**0.75 [0.71–0.79]**
	Partial r								–
Mean		73.06	0.49	3.47	3.38	4.06	5.02	3.83	2.59
*SD*		8.75	0.50	0.53	0.48	0.48	0.83	0.44	0.53

Note*.* Partial correlations controlled for age and gender; the bold numbers on the diagonal are the Intraclass Correlation Coefficients with 95% confidence interval

These results indicated that not gender and age but wellbeing and character strengths are significant variables in explaining suicidal ideation among older adults. Moreover, low SD indicated the homogeneity of the investigated population.

### Hierarchical regression analysis

Collinearity statistics indicated that all VIF values were less than 3; thus, multicollinearity was not an issue among the predictors in the present data. The results were shown in step 1, that is, the gender and age factors did not significantly contribute to the variance of suicidal ideation (*t* = − 0.16 and − 0.88, *p =* 0.87 and 0.83, respectively). In step 2, the wellbeing, including life satisfaction and thriving, explained the observed 36.3% variance of suicidal ideation in the nursing home sample (*B* = − 0.14 and − 0.52, *t* = − 2.55 and − 9.64, *p =* 0.01 and *p <* 0.001, respectively). However, in step 3, the wellbeing, life satisfaction, and thriving did not significantly account for the additional variance of suicidal ideation (*t* = 1.07 and − 0.93, *p =* 0.28 and 0.35, respectively) after character strengths and demographics were controlled. Notably, the three character strengths, caring (*B* = − 0.45, *t* = − 11.84, *p <* 0.001), inquisitiveness (*B* = − 0.33, *t* = − 7.89, *p <* 0.001), and self-control (*B* = − 0.45, *t* = − 10.83, *p <* 0.001), were significant contributors that explained a total of 65.1% variance of suicidal ideation, while controlling for wellbeing and demographic variables. Table 3 shows the results.

**Table 3:** Hierarchical regressions of demographic variables, character strengths, and wellbeing on suicidal ideation (*N* = 308)

Independent Variables	Dependent Variable: Suicidal ideation								
	Step 1			Step 2			Step3		
	*Beta*	*t*	VIF	*Beta*	*t*	VIF	*Beta*	*t*	VIF
Gender	−0.01	−0.16	1.01	−0.02	−0.44	1.01	−0.01	−0.26	1.02
Age	−0.05	−0.88	1.01	−0.04	− 0.86	1.01	− 0.04	−1.02	1.01
Life Satisfaction				−0.14*	−2.55*	1.39	0.05	1.07	1.56
Thriving				−0.52***	−9.64***	1.38	−0.05	− 0.93	2.23
Self-control							−0.45***	−10.83***	1.23
Inquisitiveness							−0.33***	−7.89***	1.48
Caring							−0.45***	−11.84***	1.50
*R*^*2*^	0.03			0.365***			.651***		
Δ *R*^*2*^				0.363***			.286***		
Δ *F*				86.604***			81.951***		

* *p* < .05; ** *p* < .01; *** *p* < .001

## Discussion

In this study, we examined the psychometric properties of TICS with a sample of 308 older adults aged at least 50 years old and the possible protective factors (i.e., character strengths and wellbeing) of suicidal ideation. TICS was found to be consistent in measuring the three character strengths and showed a cross-sectional relationship with suicidal ideation among the older adults.

Results from the second part showed that, contrary to our expectations, wellbeing, including life satisfaction and thriving, was insignificantly related to suicidal ideation after controlling character strengths. This result was inconsistent with multiple previous research studies, which indicated a direct relationship between wellbeing and suicide risk in the healthy and unhealthy population [5, 45]. Notably, character strengths showed an independent association with suicide ideation beyond the effect of wellbeing, explaining a total of 65.1% variance of suicidal ideation in this research. Cognitive factors play a significant role in affecting the suicidal ideation of older adults. Moreover, individuals likely have different perceptions of their wellbeing conditions and different levels of occurrence of suicide ideation even when facing the same circumstances due to the influence of cognition (e.g., cognitive aging, cognitive bias, and appraisal styles). For example, high levels of cognitive functioning have been found to be robustly related to better psychological wellbeing among older adults [46, 47]. By contrast, at a decreased cognitive functioning level, older adults likely experience severe psychological pain (e.g., depression and hopelessness), which increases their risk of suicidal ideation [48]. Moreover, depressed individuals with negative beliefs and cognitive biases (e.g., biased self-referent cognitive style and hopelessness) showed a high level of suicide ideation [49]. Moreover, positive self-appraisals, functioning as a resilient factor, may buffer individuals from suicidal ideation [50, 51]. Character strengths play the role of a cognitive schema, serving to organize categories of information related to oneself, others, and the world [52]. Considerable research findings, including ours, have demonstrated the positive relationship between character strengths and wellbeing across culture, lifespan, and population [25, 26, 32, 53, 54]. Given the same conditions, older people with high character strengths likely perceive difficult moments in life in a less negative manner than those with low character strengths. In this way, they may enjoy better wellbeing and less likely generate suicidal ideation. Therefore, this finding may explain why personal protective factors, namely, character strengths, presented a high percentage of variance (65.1%) of suicide ideation after controlling the effect of social protective factors, namely, wellbeing. However, further studies are necessary. Moreover, in the complete model, life satisfaction did not contribute to the variance of suicidal ideation. Among older adults, particularly those living in nursing homes, a significant difference exists between global life satisfaction, as evaluated by SWLS (which measures one dimension of wellbeing and is a cognitive evaluation or judgment of one’s life), and satisfaction with present life. Therefore, questions in SWLS may have different implications for elderly persons; they may be pleased with the lives they have led but unsatisfied with their current lives.

Among character strengths, a significant negative association was found between the character strength of self-control and suicidal ideation. Our findings were consistent with several studies between westerners and easterners that comprise certain relationships between self-control and suicide. For example, previous studies have proposed that suicide attempts and poor psychological functioning are related [55]. Depressed patients had higher scores on maladaptive schemas, such as recognition seeking, negativity/pessimism, and insufficient self-control, than healthy controls [55]. This finding shows that in different groups, self-control has a significant effect on suicidal ideation or attempts. Moreover, by using the data from the National Survey of Child and Adolescent Wellbeing, researchers observed that self-control predicts suicidal tendencies [56]. The effect of self-control remains significant when controlling for depression in juveniles and previously reported suicidal thoughts and behaviors [56]. Our findings determined that character strengths accounted for a significant additional variance in suicide ideation, which was consistent with this study to a certain degree. Furthermore, previous studies have demonstrated that the effects of self-control on wellbeing and psychosocial adjustment are significant [57, 58]. Therefore, when facing stress and adversity, self-control may promote wellbeing and positive adaptation for older adults [58], which may prevent them from suicide ideation [59].

Second, a high level of the character strength of caring was significantly associated with a low level of suicide ideation among older adults in our study. Although research on a direct relationship between caring and elderly people’s suicidal ideation is limited, one study indicated that caring and connections are important protective factors for adolescent health and wellbeing [60]. Although we did not analyze the influences of different kinds of caring on suicidal ideation, multiple previous research studies explored the relationship between gratitude and suicidal ideation. One research indicated that gratitude was closely associated with less suicidal ideation and suicide attempts among Chinese adolescents [61]. Another research also indicated that gratitude is a protective factor that mediated the negative relationship between humor and suicidal ideation [62]. Our result is consistent with that of previous ones. Phillip Slater [63] noted that the desire for the community, engagement, and dependence are deeply human desires that can create meaning and happiness. Moreover, the thwarted belongingness has been identified as proximal causes of suicidal ideation [2, 5]. Furthermore, older adults who presented agreeableness and conscientiousness likely perceive positive relationships with others and high psychological wellbeing [64], and likely generate suicidal ideation [59]. This notion can be one possible explanation of why caring can be a powerful human strength that plays a role against negative events in life and protect people from suicidal ideation.

Third, the character strength of inquisitiveness was also found to present a significant negative relationship with suicidal ideation among older adults. Numerous previous studies have identified the positive relationship between inquisitiveness (e.g., curiosity and zest) and psychological wellbeing [65, 66]. This relationship has been examined as an important protective factor against suicidal ideation and behaviors [5, 45]. Moreover, high trait curiosity was significantly associated with a lower rate of mortality among the aging population [67]. A recent study directly presented that high curiosity could predict the increased coping efficacy in stopping negative thoughts over time in veterans with suicidal ideation [68]. These findings, including ours, suggested that elderly people with a high level of inquisitiveness have lower tendencies to generate suicidal ideas. One possible reason is that with the character strength of inquisitiveness, older adults likely develop and maintain flexible and adaptive responses to age-related challenges, thereby preventing them from suicidal risks [67].

Last, our results indicated that gender was insignificantly associated with suicidal ideation in the current sample, which was inconsistent with previous studies claiming that the Chinese female group experience a greater risk of suicide than the male group [69, 70]. However, a recent study has demonstrated a downward trend of national male–female differences in elderly suicide rates in China [71]. One possible reason lies in the marked magnitude of differences between urban and rural areas [72]. Rural male–female differences among the elderly contributed most to the national male–female differences in China [71]. The differences between male–female suicide rates in rural areas have been decreasing. Thus, the national male–female differences reduced, whereas the urban male–female differences increased [71]. However, further examinations are needed.

Suicide rates are associated with suicidal ideation in older adults, but research on preventive interventions in this age group is limited [73]. Therefore, the results of this study may be of practical significance, which can enrich the existing research theories and provide theoretical support for subsequent prevention and intervention efforts. Our findings indicated that character strengths were cross-sectionally related to suicidal ideation. Self-control, caring, and inquisitiveness were associated with low levels of suicidal ideation. Therefore, as protective factors of suicidal ideation, strategies that implement certain activities or practices related to character strengths can be used through preventive interventions. Prevention of suicidal ideation should incorporate integrative perspectives to create a series of targeted and indicated interventions in nursing homes, communities, hospitals, and individuals. Strategies should focus on strengthening the character strengths of elderly people to modify their thought trajectory and prevent them from generating suicidal ideation. Thus, further research and application to a wide range of groups for preventing suicidal ideation in older adults in China are timely and necessary.

### Limitations

The findings must be interpreted in light of several limitations. First, the primary limitation resides in the cross-sectional nature of the study. The cross-sectional study design prevents us from detecting a direct causal relationship between the nominated possible protective factors in our study and the suicide ideation.

Second, the generalizability of the findings can be regarded as another limitation of the present study. The sample in the study is not a nationwide sample, and only older adults in nursing homes of Chongqing are selected as research subjects. However, Chongqing, with a medium level of development, may have a certain degree of representation. In addition, the current study collected data at nursing homes, which limits the representative of the population. For example, we used the exclusion criteria, such as not diagnosed with depression, to select subjects in this study. Although not having a diagnosis of depression does not mean that the participant is not clinically depressed, we cannot easily generalize our findings to the clinical sample. Some researchers have determined that no direct link exists between depression and suicidality in nonclinical populations [74]. However, depression was considered an important factor influencing suicidal ideation [8]. Therefore, the result of this study cannot be applied to the clinical population with depression.

Third, the sampling and recruitment procedure may influence the results. Allowing the social workers to exclude certain older adults from the recruitment implied the potential to generate a selection bias in the sample, which, though, would be helpful to guarantee that all eligible participants had the basic abilities (e.g., understanding the questions and responding to them) to complete the questionnaire without major difficulties. Moreover, the exclusion criteria should include the acute risk of suicide because those who were at acute risk for suicide would be under significant stressors, have a high level of suicide ideation, and require immediate intervention [75]. The means of convenience sampling was also likely to be biased. In contemporary China, nursing homes are newly developed because of tremendous social change. The traditional family-based support system alone was inadequate to cope with the rapid social change and the increase in the aged population due to the one-child policy in China. Children are busy with work, usually in another city, and therefore have no time to take care of their older parents. Therefore, older adults chose to live in nursing homes. However, some older adults, for example, those who have limited access to nursing homes or living in the community, were excluded from the study sample.

The psychometric properties of the measurements were a noteworthy limitation. The measures we used in this study may have the issue of cross-cultural variations, which influenced the level of reliability. For example, regarding the measures of wellbeing, the concept of “life satisfaction” has been demonstrated to be similarly understood across a wide range of cultures, whereas the Satisfaction with Life Scale showed metric equivalence in different cultures [76–78]. The Cronbach’s alpha (similar to the ICC value) is 0.76 in the current sample, which is similar to the older adults from France (0.80) [79], Mexico (0.74) [80], and Ecuador (0.79) [81]. However, the cross-cultural variations of thriving are not well discussed in existing studies. The BIT showed relatively high reliability in older American adults (Cronbach’s alpha = 0.90) [37], whereas the level of reliability is moderate in the current Chinese elderly population (ICC = 0.69). These results indicated that people from different cultures may hold an inconsistent reaction to the concept of “thriving,” which could influence the psychometric properties of BIT in different language versions. Specifically, the emphasis on filial piety (*Xiaodao*) is rooted in Chinese culture for centuries. The intergenerational co-residence and taking care of parents remain an important obligation of children. These actions are also signs of respect and social support for the older generation, which may directly protect the mental health and wellbeing of older Chinese parents [82–85]. Therefore, our participants may rate lower scores on certain items of BIT (e.g., *I feel a sense of belonging in my community; There are people who appreciate me as a person*), which reduce the internal consistency of BIT. Additionally, the living experience and conditions of the participants influence the perception of character strengths. A recent study has demonstrated that demographics (e.g., age, marital status, and retirement status) and living conditions (e.g., living with families and partners) are associated with an individual’s character strengths [86]. For example, the strengths of curiosity are positively related to the living conditions (i.e., living together), whereas the strengths of creativity are negatively associated with the living conditions [86]. All participants in our study lived in the nursing house, separated from their families (e.g., children). Therefore, they may rate lower scores on the item of curiosity but higher scores on the creativity, which may cause certain effects on the internal consistency of the character strengths of inquisitiveness. Moreover, compared with the character strengths of caring, the Cronbach’s alpha of the inquisitiveness has been relatively lower in the surgery inpatient sample [24], who do not live with their families.

Another influencing issue is the length of the instruments. The relatively low level of reliability of TICS and BIT may be associated with the briefness of TICS and BIT as the coefficient (e.g., α), which can be strongly affected by the length of the measurements’ scale [87]. Using Cronbach’s alpha as the indicator may mislead to an impression of poor reliability when using brief scales [27, 44]. One previous research using Ryff’s Scales of Psychological Wellbeing, which comprises 18 questions for six factors, found that the lowest Cronbach’s alpha estimate of one dimension was 0.53 [88]. This finding may indicate that BIT (containing only 10 items) and TICS of inquisitiveness and self-control (each containing five items) can be acceptable. However, the starting level of 0.70 is suggested.

To find additional convincing evidence, future studies should use a multi-wave longitudinal design to investigate the relationship among character strengths, wellbeing, and suicide ideation over time among a generalizable community or clinical samples. Random samples in different circumstances should be used in future studies to reduce the selection bias. The recruitment procedure could also be enhanced by conducting a suicide risk assessment, which is helpful in expanding this work to older adults under different categories of suicide risks (e.g., acute and chronic suicide risks), who may report different levels of previous suicide ideation, planning, and attempts [75]. More studies should be conducted to investigate the psychometric properties of the measurements (e.g., life satisfaction, thriving, and character strengths) before applying the instruments. Specifically, further studies should be conducted to test whether the items in different languages could achieve an equivalent meaning, whether the same concept and construct represent the same behaviors and values in different cultures among different groups, and whether the psychometric properties (e.g., reliability and validity) are of the same measure in various cultures and populations.

## Conclusion

In summary, we showed that TICS could be used to assess the character strengths among older adults. Character strengths were associated with low levels of suicidal ideation. As the research on interventions to reduce suicidal ideation and behaviors in older people is limited, we believe that future studies should accumulate similar evidence and provide theoretical support for subsequent interventions.

## Data Availability

The datasets used and/or analysed during the current study are available from the corresponding author on reasonable request.

## Abbreviations

BIT: Brief Inventory of Thriving
CFI: Comparative Fit Index
ESEM: Exploratory Structural Equation Modelling
GSIS: Geriatric Suicide Ideation Scale
RMSEA: Root Mean Square Error of Approximation
SWLS: Satisfaction with Life Scale
TICS: Three-Dimensional Inventory of Character Strengths
TLI: Tucker–Lewis Index
VIA-IS: Values in Action Inventory of Strengths
VIF: Variance Inflation Factor
